# Lovastatin Induces Multiple Stress Pathways Including LKB1/AMPK Activation That Regulate Its Cytotoxic Effects in Squamous Cell Carcinoma Cells

**DOI:** 10.1371/journal.pone.0046055

**Published:** 2012-09-28

**Authors:** Laurie Ma, Nima Niknejad, Ivan Gorn-Hondermann, Khalil Dayekh, Jim Dimitroulakos

**Affiliations:** 1 Centre for Cancer Therapeutics, The Ottawa Hospital Research Institute, Ontario, Canada; 2 The Faculty of Medicine and the Department of Biochemistry, University of Ottawa, Ontario, Canada; UAE University, United Arab Emirates

## Abstract

**Background:**

Cellular stress responses trigger signaling cascades that inhibit proliferation and protein translation to help alleviate the stress or if the stress cannot be overcome induce apoptosis. In recent studies, we demonstrated the ability of lovastatin, an inhibitor of mevalonate synthesis, to induce the Integrated Stress Response as well as inhibiting epidermal growth factor receptor (EGFR) activation.

**Methodology/Principal Findings:**

In this study, we evaluated the effects of lovastatin on the activity of the LKB1/AMPK pathway that is activated upon cellular energy shortage and can interact with the above pathways. In the squamous cell carcinoma (SCC) cell lines SCC9 and SCC25, lovastatin treatment (1–25 µM, 24 hrs) induced LKB1 and AMPK activation similar to metformin (1–10 mM, 24 hrs), a known inducer of this pathway. Lovastatin treatment impaired mitochondrial function and also decreased cellular ADP/ATP ratios, common triggers of LKB1/AMPK activation. The cytotoxic effects of lovastatin were attenuated in LKB1 null MEFs indicating a role for this pathway in regulating lovastatin-induced cytotoxicity. Of clinical relevance, lovastatin induces synergistic cytotoxicity in combination with the EGFR inhibitor gefitinib. In LKB1 deficient (A549, HeLa) and expressing (SCC9, SCC25) cell lines, metformin enhanced gefitinib cytotoxicity only in LKB1 expressing cell lines while both groups showed synergistic cytotoxic effects with lovastatin treatments. Furthermore, the combination of lovastatin with gefitinib induced a potent apoptotic response without significant induction of autophagy that is often induced during metabolic stress inhibiting cell death.

**Conclusion/Significance:**

Thus, targeting multiple metabolic stress pathways including the LKB1/AMPK pathway enhances lovastatin’s ability to synergize with gefitinib in SCC cells.

## Introduction

A fundamental requirement of all cells is the ability to adapt to changes in their environment that includes the availability of nutrients required to maintain homeostasis or to trigger proliferation only when they are in sufficient supply [1], [2]. Under conditions of metabolic stress where nutrients are limited, a number of cellular stress pathways can be activated that attempt to alleviate this stress or if the stress cannot be overcome to induce apoptosis [3], [4], [5]. Common downstream targets of these pathways include the inhibition of protein translation, which is the major energy consumption process within cells, and the induction of cell cycle arrest to prevent cell proliferation [5], [6], [7]. Glucose deprivation, hypoxia, amino acid starvation, growth factor withdrawal and conditions or agents that affect ATP synthesis are all common triggers of metabolic stress [1], [2]. A number of signaling pathways regulate the cellular response to these stressors including the Integrated Stress Response (ISR) [5], [8], growth factor signaling [9], [10] and the Liver Kinase B1 (LKB1)/AMP-Activated Protein Kinase (AMPK) pathway that is a sensor of cellular energy (ATP) levels [3], [6], [11].

Malignant cells are dependent on the sustained availability of the end products of the mevalonate pathway that include de novo cholesterol and isoprenoids [12], [13]. The statin family of drugs are potent inhibitors of 3-hydroxy-3-methyl glutaryl coenzyme A (HMG-CoA) reductase that bind to the active site of HMG-CoA reductase with up to a 1000x higher affinity than its natural substrate [14]. Through inhibition of mevalonate synthesis, statin treatment has the potential for plieotropic cellular effects through the targeting of a wide variety of cell signaling proteins [12], [13], [15]. We have previously shown that lovastatin can induce tumour specific apoptosis especially in squamous cell carcinomas (SCC) [16] and that 23% of SCC patients treated with lovastatin as a single agent showed disease stabilization in our Phase I clinical trial [17]. To enhance efficacy, understanding the mechanism of lovastatin-induced apoptosis may uncover novel therapeutic strategies.

To this end, we have recently demonstrated that the ability of lovastatin to induce metabolic stress responses in SCC cells through the induction of the ISR [18] and inhibition of ligand induced activation of the epidermal growth factor receptor (EGFR) [19]. Both pathways play key roles in regulating lovastatin-induced apoptosis in SCC cells. A variety of defined cell stresses commonly lead to the phosphorylation of eukaryotic initiation factor (eIF) 2α shutting down global protein translation and inhibiting cell proliferation to potentially alleviate the stress known as the ISR [5]. The α subunit of eIF2 is the target of a family of serine or threonine kinases that are activated by different forms of environmental and metabolic stresses that includes the General Control Non-repressed 2 (GCN2) gene induced by amino acid starvation [5]. Depending on the strength of the stress stimulus, ISR induction can either alleviate the stress or alternatively induce apoptosis. Enhanced expression of the transcription factors activating transcription factor (ATF) ATF3 and C/EBP-homologue protein (CHOP) can mediate this apoptotic response [5], [20].

Activation of the EGFR in epithelial cells and their derived tumours including SCC is stimulated by ligand binding that is required to activate its downstream signaling targets that mediate its effects on growth, metabolic activity and cell survival [9], [10], [21]. EGFR autophosphorylation sites when activated by ligand binding are the biochemical triggers that start a series of downstream signaling cascades that regulates these effects of this receptor including the activation of the phosphatidylinositol-3 kinase (PI3K)/AKT pathways [10]. Besides promoting cell survival, signaling by the PI3K/AKT pathway also affects mRNA translation through the activation of mTOR (mammalian target of rapamycin) and the subsequent phosphorylation of eukaryotic translation initiation factor 4E binding protein 1 (4EBP1) [22], [23]. Hyperphosphorylated 4EBP1 is released from eukaryotic translation initiation factor 4E (eIF4E) resulting in enhanced cap-dependent translation [22], [23]. mTOR is a serine/threonine kinase that is conserved in eukaryotes and is a central regulator of cell proliferation that is active under nutrient-rich conditions and growth factor activation and its activation requires positive signals from both nutrients (glucose, amino acids) and growth factors [24]. In SCC cells, we demonstrated that lovastatin inhibits ligand induced EGFR and AKT activation and 4EBP1 phosphorylation [19].

Thus, we have demonstrated the ability of statins to target multiple pathways that mediate metabolic stress and that these pathways regulate statin-induced tumour cell apoptosis [18], [19], [25]. mTOR and 4EBP1 as well as HMG-CoA reductase activities are also regulated by AMPK [26]. AMPK is a sensor of cellular energy status that is activated under conditions of low intracellular ATP associated with nutrient deprivation or hypoxia [27]. AMPK is a highly conserved heterotrimeric kinase complex composed of a catalytic (α) subunit and two regulatory (β and γ) subunits [27]. AMPK is activated when intracellular ATP levels decline and intracellular AMP increases as AMP directly binds to the AMPK γ subunit preventing dephosphorylation of the critical activation loop threonine in the α subunit [28]. The serine/threonine kinase LKB1 represents the major kinase phosphorylating the AMPK activation loop under conditions of energy stress [11]. Consistent with a role for AMPK in growth control, its major upstream kinase LKB1 is a human tumor suppressor [29]. LKB1 mutations are prevalent in a large percentage (30–40%) of sporadic non-small cell lung cancers (NSCLC) as well as other epithelial derived cancers [29]. Cellular distribution of LKB1 is mediated by co-factors STRADα and MO25 where STRADα induces its re-localization from the nucleus to the cytoplasm and MO25 stabilizes this interaction stimulating LKB1 catalytic activity including activation of AMPK [30]. Of clinical significance, the drug metformin, that is used to treat type-2 diabetes, is a potent inducer of LKB1 and is currently being evaluated as an anti-cancer therapeutic agent [31].

In this study, we evaluated the potential of lovastatin treatment to induce LKB1 and AMPK activity in concert with our previously identified ability of lovastatin to activate the ISR and inhibit EGFR/AKT activation in SCC cells. The ability of statins to induce multiple metabolic stress pathways may have clinical relevance regulating statins ability to synergize with EGFR kinase inhibitors in SCC cells.

## Materials and Methods

### Tissue Culture

The human tumor derived cell lines SCC9, SCC25 (head and neck carcinomas) and HeLa (cervical carcinoma) as well as A549 (lung carcinoma) were obtained from the ATCC (Rockville, MD, USA). The murine embryonic fibroblasts (MEFs)/LKB1^−/−^ were kindly provided by Dr. R. Screaton (Research Institute, Children’s Hospital of Eastern Ontario, Ottawa, Canada) [32]. Cells were maintained in Dulbecco’s-MEM (Media Services, Ottawa Regional Cancer Centre) supplemented with 10% fetal bovine serum (Medicorp, Montreal, QC, Canada). Based on the manufacterer’s description, the complete media with FBS would possess a concentration of 37 µg/ml cholesterol compared to greater than 100 µg/ml typically present in human serum [33], [34]. Cells were exposed to solvent control, or 0–100 µM lovastatin (generously provided by Apotex, Mississauga, Canada; 10 mM stock in ethanol), 0–100 µM gefitinib (kindly provided by AstraZeneca Macclesfield, England, 10 mM stock in DMSO), 50 µg/ml human recombinant EGF (Sigma, St. Louis, MI, USA, 10 mg/ml stock in 10 mM acetic acid/0.1% bovine serum albumin). Cells were also treated with AMPK activators, 0–20 mM metformin (Sigma, 500 mM stock in ddH_2_O) and 0–1 mM AICR (Sigma, 100 mM stock in ddH_2_O); and 0–5 µM AMPK inhibitor, compound C (Calbiochem, San Diego, CA, USA, 10 mM stock in DMSO).

**Figure 1:**
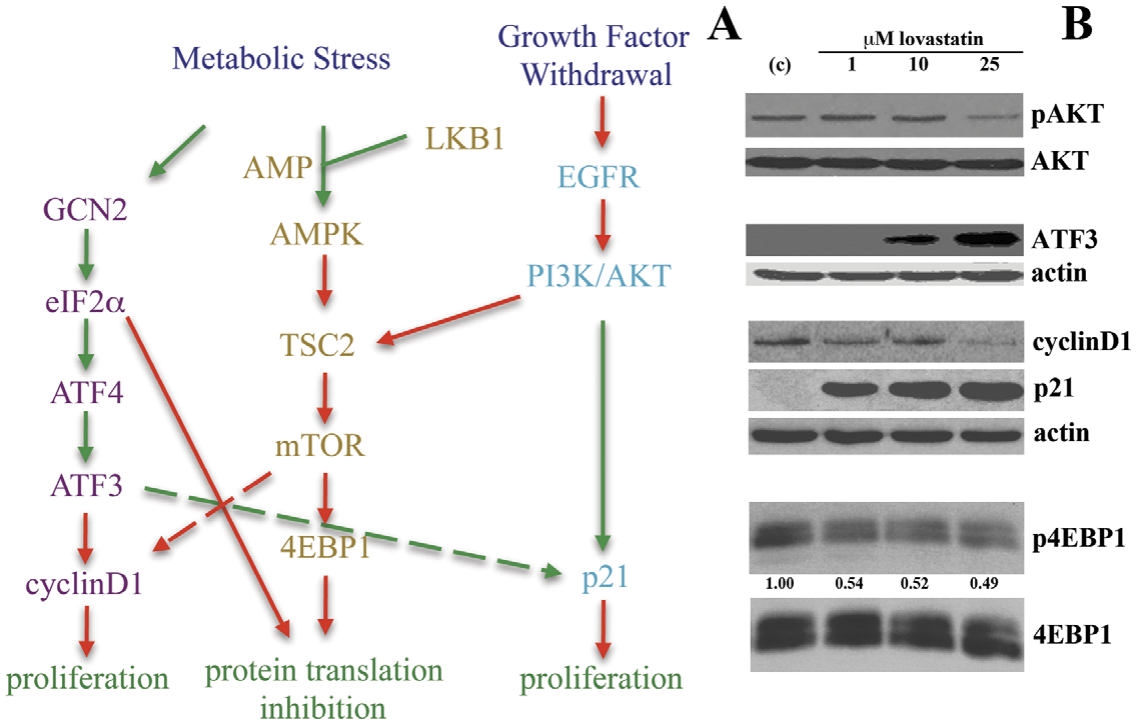
A number of interacting signaling pathways regulate metabolic and growth factor withdrawal cellular stress responses. *(A)* Lovastatin-induced apoptosis is regulated in part by the inhibition of the epidermal growth factor receptor (EGFR) mimicking growth factor withdrawal and the induction of the Integrated Stress Response (ISR) through induction of GCN2. In this study, we evaluated the potential of lovastatin to activate the LKB1/AMPK pathway that is induced upon ATP depletion. *(B)* Western blot analysis of SCC25 cells treated for 24 hrs showing lovastatin regulation of downstream targets of the EGFR (pAKT), the ISR (ATF3) and common targets of all three pathways including the cell cycle regulators cyclinD1and p21 and a regulator of protein translation, 4EBP1. Densitometric analysis of 4EBP1 is presented where the ratio of p4EBP1 to 4EBP1 expression was determined with the control untreated SCC25 cell line value represented as 1.00 for ease of presentation.

### Western Blot Analysis

Cell pellets were lysed for 30 min in lysis buffer that contained 10 mM Tris-HCI (Sigma), pH 7.4, 150 mM NaCI (Sigma), 5 mM EDTA (Sigma), 1% Trion X-100 (Sigma), 1 mM sodium orthovanadate (Sigma), 5 mM sodium fluoride (Sigma) and protease inhibitor cocktail (Sigma). Total protein was quantified with the Biorad Protein Assay using bovine serum albumin (Sigma) as standard. Protein extracts representing 50 µg total protein were subjected to electrophoresis on 10% SDS-PAGE gels. Proteins were transferred to polyvinylidene difluoride membranes (Millipore, Billerica, MA, USA) and the membranes were probed with antibodies from Cell Signaling Technology (Danvers, MA, USA) including anti-LKB1 and phospho-LKB1 (Ser428), anti-AMPK and phospho-AMPK (Thr172), anti-Akt and phospho-Akt (Ser473), anti-4EBP1 and phospho-4EBP1 (Thr37/40), and antibodies from Santa Cruz including anti-ATF3, anti-p21, anti-cyclinD1 and anti-actin. The immunoblots were developed by ECL (Thermo Scientific, Vernon Hills, IL, USA). The images were acquired using the Gene Gnome Imaging System (Syngene Bio-imaging, Frederick, MD, USA).

**Figure 2:**
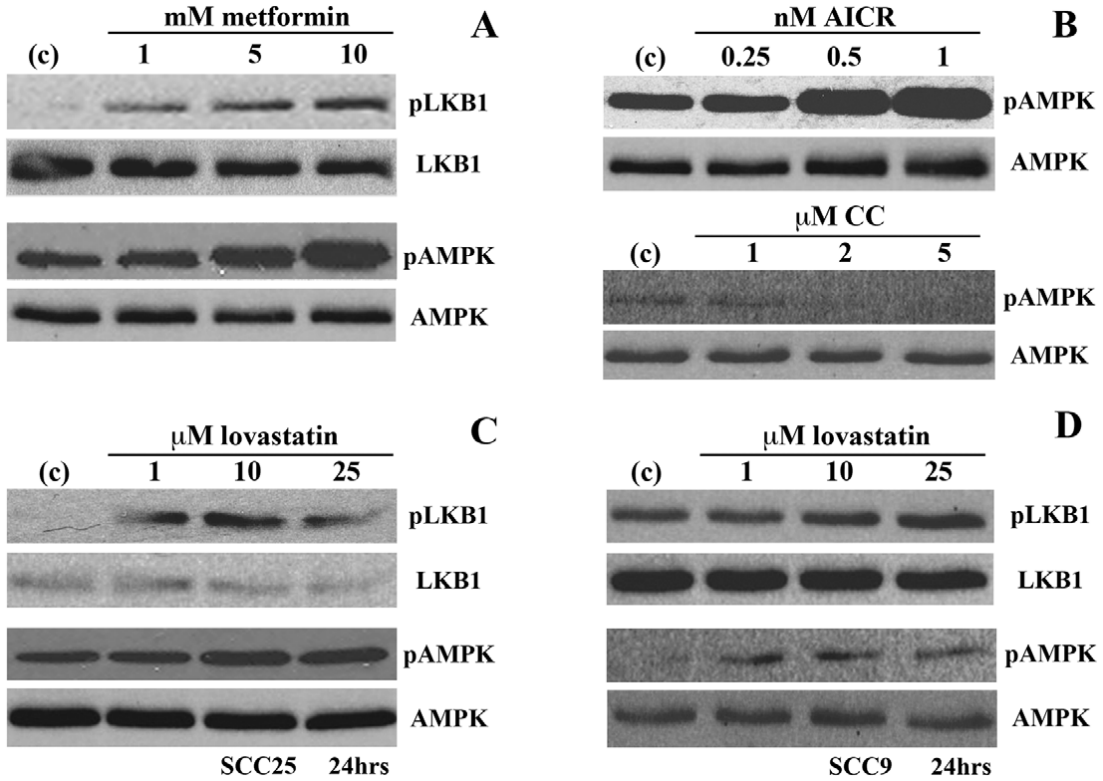
Lovastatin treatment induces activation of LKB1 and AMPK in SCC cells. *(A* and *B)* Western blot analysis of activated phosphorylated LKB1 (ser428) and AMPK (thr172) along with total LKB1 and AMPK as loading controls in SCC25 treated with known activators of this pathway, metformin and AICR, and an inhibitor compound C (CC). (*C and D)* Similar Western blot analyses of treatments of SCC9 and SCC25 cells with 0–25 µM lovastatin for 24 hrs demonstrated a dose dependant induction of activated LKB1 and AMPK.

### Immunofluorescence

In a 6-well flat-bottomed plate (Fisher, Mississauga, ON, Canada), glass cover slips (Fisher) were placed into each well and ∼250000 cells were used to seed each well. The cells were incubated overnight to allow for cell attachment and recovery. Following a 24-hour treatment of solvent control or lovastatin, the cells were subsequently washed with PBS then fixed with 4% paraformaldehyde (Sigma) buffered in PBS for 15 minutes at 37°C and stored in PBS at 4°C. Prior to immunofluorescence staining, the cells were permeabilized with PBS+0.2%Triton X-100 (Sigma) for 15 minutes. The cells were blocked for 30 minutes with PBS+3%FBS then incubated with the cytochrome c antibody (Pharmingen, San Diego, CA, USA) at a dilution of 1∶50 in PBS+3%FBS for an hour. The cells were then blocked with PBS+5%FBS for 30 minutes. Following the second blocking, the cells were incubated with Alexa Fluor 488 goat anti-mouse IgG (Molecular Probes, Carlsbad, CA, USA) at a working dilution of 10 µg/ml in the dark for an hour. The cells were then mounted to a microslide with DAPI mounting media (Vector Laboratories, Burlingame, CA, USA) and analyzed under fluorescent microscopy using the Axiovision software (Allied High Tech Products, Rancho Dominguez, CA, USA). For visualization of the green fluorescent protein (GFP) chimeras used in this study included GFP-LKB1 in the GFPc2 plasmid backbone (Addgene, Cambridge, MA, USA) and LC3-GFP in the pCMV plasmid backbone kindly provided by Dr. Xiaohui Zha (Ottawa Hospital Research Institute, Ottawa, Canada). Plasmids were transfected and expressed in cells employing Lipofectamine (Invitrogen, Carlsbad, CA, USA) following manufacturer’s protocol, fixed and visualized using microscopy as outlined above.

**Figure 3:**
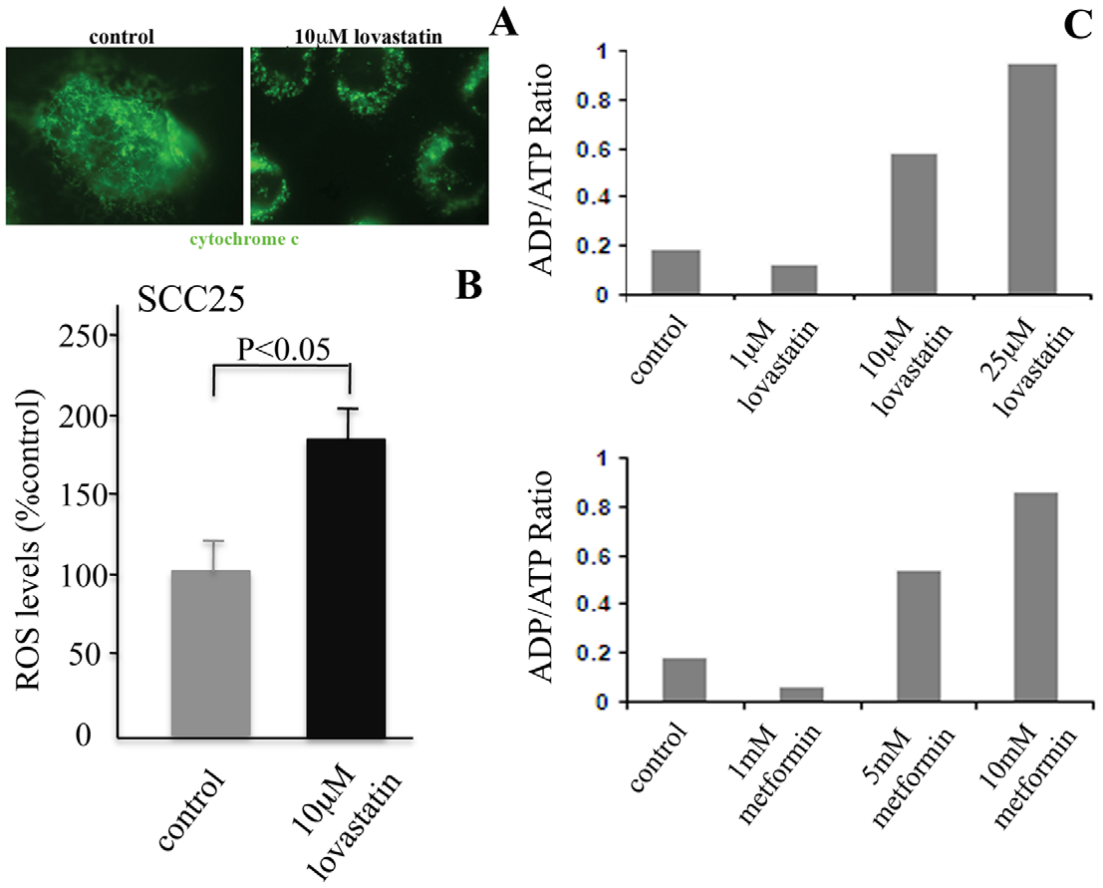
Lovastatin treatment affects ATP levels in SCC25 cells. *(A)* Mitochondrial morphology was assessed by immunofluorescent localization of cytochrome c, an important mitochondrial resident protein. In control cells, cytochrome c was expressed in tube-like structures typical of mitochondrial distribution pattern. In SCC25 cells treated with 10 µM lovastatin for 24 hrs, cytochrome c staining was more punctate staining indicative of mitochondrial fragmentation, a common feature under conditions of mitochondrial-mediated apoptosis. *(B)* Reactive oxygen species (ROS) generation is an early marker of mitochondria dysfunction. We evaluated cellular ROS production by using an oxidation-based fluorescence assay and found that ROS production was increased in SCC25 cells following 24 hr 10 µM lovastatin treatment (p<0.05). *(C)* Cellular ATP levels coincident with 0, 1, 10 and 25 µM lovastatin and as a comparator with 0, 1, 5, 10 mM metformin treatments for 24 hrs in SCC25 cells were determined. The ADP:ATP ratio was determined for each condition tested using the Enzylight ADP/ATP ratio assay kit. An increased ADP:ATP ratio, reflective of declining ATP levels and increased AMP levels was observed in a dose dependant manner for both lovastatin and metformin treatments.

**Figure 4:**
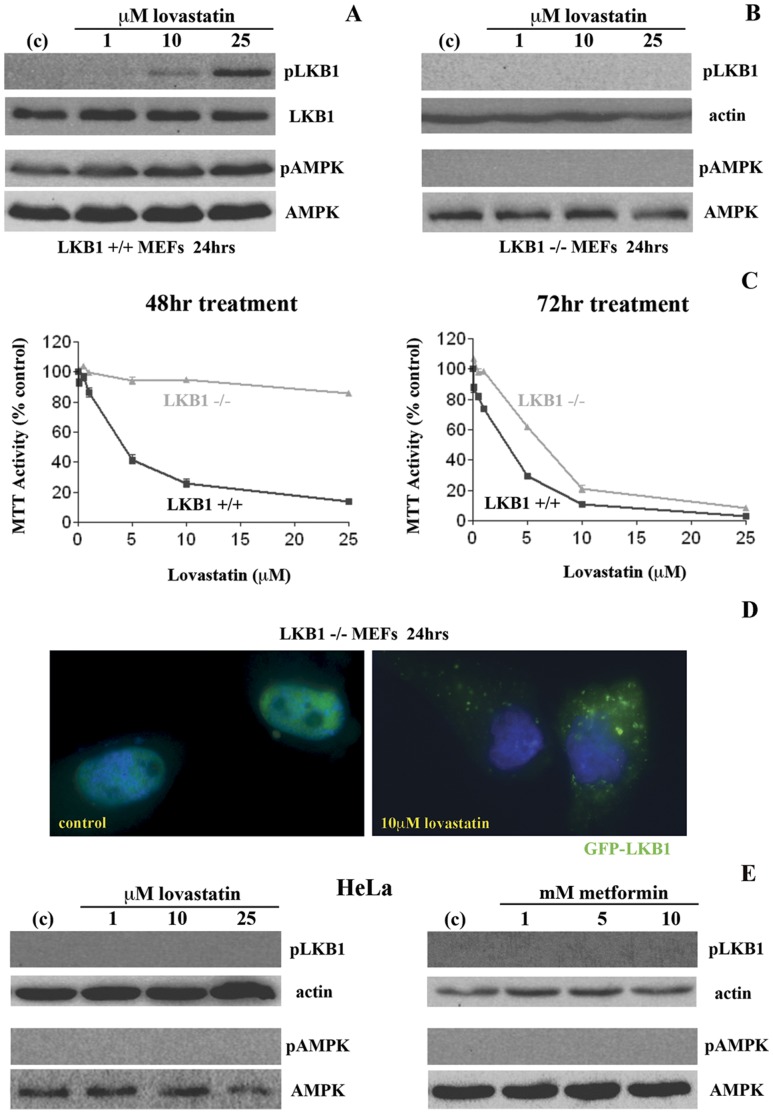
Deletion of LKB1 in MEFs inhibits lovastatin-induced cytotoxicity. *(A and B)* Western blot analysis of activated phosphorylated LKB1 (ser428) and AMPK (thr172) along with total LKB1 and AMPK as loading controls in wild-type LKB1+/+ murine embryonic fibroblasts (MEFs) compared to LKB1−/− MEFs. Lovastatin treatment (24 hr- 0, 1, 10 and 25 µM) induced phosphorylation of both LKB1 and AMPK only in the LKB1+/+ MEFs and were undetected in the LKB1−/− MEFs (due to the lack of expression of LKB1, actin was used as a loading control in the LKB1−/− MEFs). *(C)* MTT cell viability assay of LKB1+/+ and LKB1−/− MEFs treated with a range of lovastatin concentrations of up to 25 µM for 48 and 72 hrs. LKB1+/+ MEFs were significantly more sensitive to 48 hr lovastatin treatments than the LKB1−/−MEFs. However, at the 72 hr time point, the difference in cytotoxicity observed within these two sets of MEFs was not evident. *(D)* Fluorescent microscopic localization of exogenously expressed green fluorescent protein (GFP)/LKB1 chimeric protein in the LKB1−/− MEFs. In the control cells, LKB1 was exclusively localized in the nucleus of expressing LKB1−/− cells, while in the lovastatin treated cells greater than 80% of expressing cells LKB1 was localized in the cytoplasm. *(E)* Western blot analysis of activated phosphorylated LKB1 (ser428) and AMPK (thr172) along with total LKB1 and AMPK as loading controls in the human SCC cell line HeLa that is deficient in LKB1 expression. Lovastatin (24 hr- 0, 1, 10 and 25 µM) and metformin (24 hrs- 0, 1, 5, 10 mM) failed to induce the phosphorylation of AMPK (due to the lack of expression of LKB1, actin was used as a loading control).

### Reactive Oxygen Species Quantitation Assay

SCC25 cells were plated in a 6-well tissue culture dish at a density of 1.5×10^6^ cells per well. The following day cells were either treated with 10 µM lovastatin or vehicle control for 24 hrs. The cells were then washed twice with HBSS and incubated with 100 µM carboxy-H2DCFDA (Molecular Probes) in the loading medium (DMEM containing 1% FBS) for 20 mins at 37°C and washed 3 times with HBSS. DCF fluorescence was measured using Synergy Mx BioTek plate reader (Bio-Tec, Vermont, USA) with excitation/emission of wavelengths of 485/530 nm.

### ADP/ATP Ratio Determination

ATP was measured based on luciferin–luciferase reaction using Enzylight ADP/ATP ratio assay kit (ELDY-100) (BioAssay Systems, Hayword, CA, USA) as per the manufacturer’s instructions. Briefly, SCC9 and SCC25 cells (10,000 cells at 100 µl/well) were seeded in a 96-well flat-bottom plate (Costar, Corning, NY, USA) and incubated overnight to allow for cell attachment and recovery. Cells were treated with 0–25 µM lovastatin or 0–10 mM metformin for 24 hrs. After treatments, 90 µl of assay buffer that contains luciferin–luciferase (ATP dependant enzyme) was added to each well and incubated for 10 min at RT to allow the release of ATP and ADP from the cell suspensions. ATP concentration was then measured by the SyNERGY MX (Bio-Tec) reader and represented directly by the number of relative light units (RLU).

ATP + D-luciferin + O2 → oxyluciferin + AMP + PPi + light

The luminescence from the initial ATP measurement remains stable throughout this assay (RLUA). By adding 10 µl of ADP converting enzyme to each well and incubating for 10 min at RT, ADP was converted to ATP. An immediate reading was taken to determine the baseline ADP RLU (RLUB). The ratio of ADP:ATP for each well was calculated from these readings as follows:
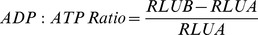



### 3-(4,5-Dimethylthiazol-2-yl)-2,5-Diphenyltetrazolium Bromide Assay (MTT Assay)

In 96 well flat bottom plates (Costar) approximately 5,000 cells/150 µl of cell suspension was used to seed each well. Cells were incubated overnight to allow for cell attachment and recovery. Following treatment, 50 µl of a 5 mg/ml solution in phosphate buffered saline of the MTT tetrazolium substrate (Sigma) was added and incubated for 6 hrs at 37°C. The resulting violet formazan precipitate was solubilized by the addition of 100 µl of a 0.01 M HCl/10% SDS (Sigma) solution shaking overnight at 37°C. The plates were then analyzed on an MRX Microplate Reader from Dynex Technologies (West Sussex, UK) at 570 nm to determine the optical density of the samples.

**Figure 5:**
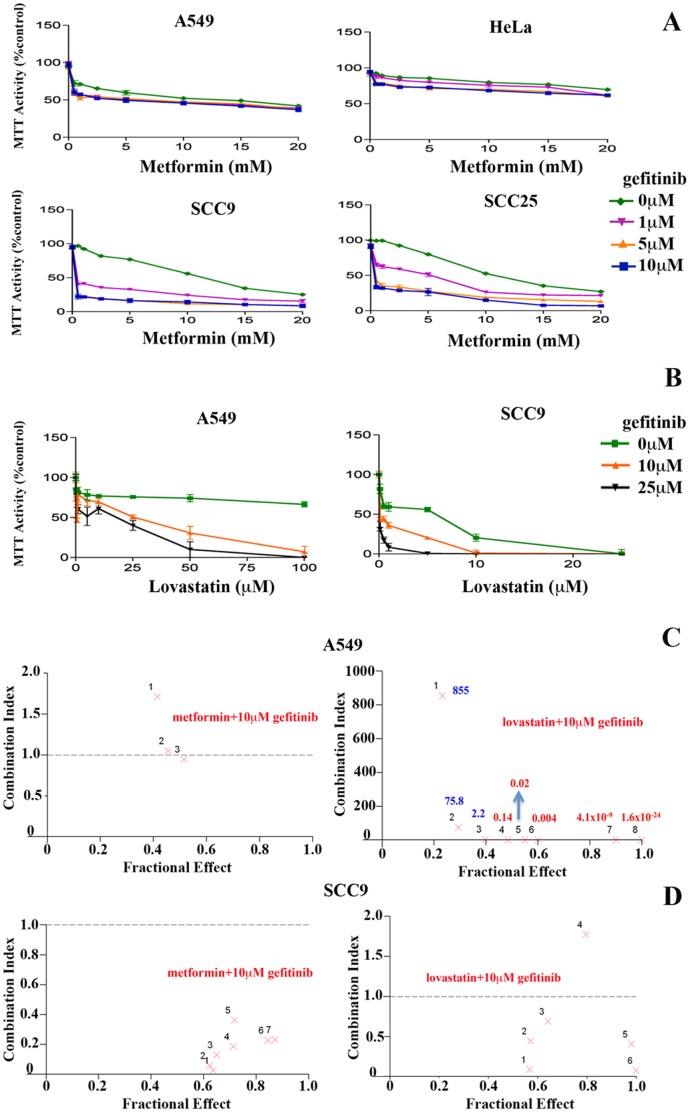
Lovastatin but not metformin can enhance the cytotoxic effects of gefitinib in LKB1 deficient tumour cells. *(A and B)* MTT cell viability assays to evaluate the potential of metformin (0–20 mM) or lovastatin (0–100 µM) treatments to enhance the cytotoxicity of gefitinib (1–25 µM) in various LKB1 deficient cell lines including A549 (NSCLC) and HeLa and LKB1 expressing SCC25 and SCC9 cell lines. Metformin and lovastatin treatments were for 72 hrs (24 hr pretreatment) while the addition of gefitinib was for 48 hrs. *(C and D)* The Chou-Talalay method was used to distinguish between antagonistic, additive and synergistic cytotoxicity in the A549 and SCC9 with the combination of agents at various fractions of cell cytotoxicity (Fractional Effect). The Combination Index (CI) values of 1 are additive, less than 1 are synergistic and more than 1 are antagonistic and determined by CalcuSyn 2.0 software analysis. In the LKB1 deficient A549 cell line, metformin in combination with 10 µM gefitinib displayed antagonism in combination, while lovastatin in combination with 10 µM gefitinib consistently displayed synergism while in the SCC9 cell lines similar combinations of metformin or lovastatin with 10 µM gefitinib both displayed synergy in combination.

**Figure 6:**
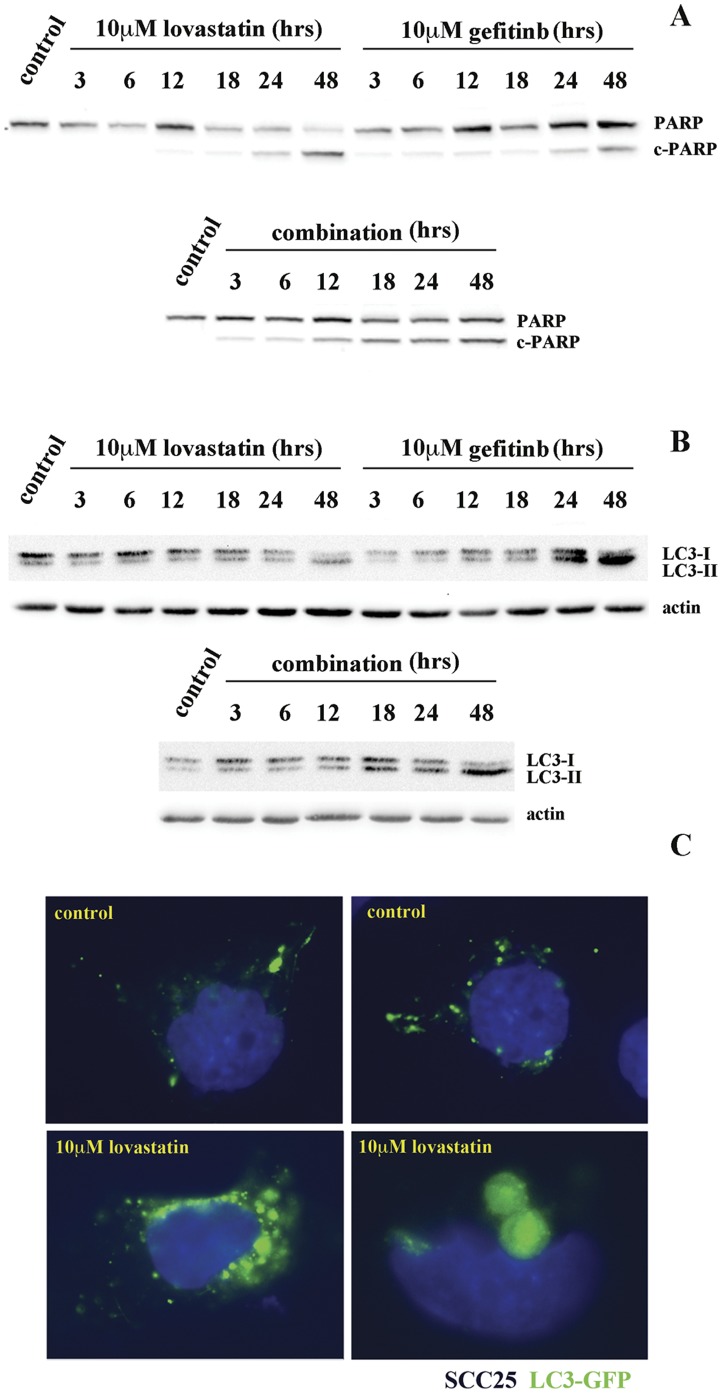
Lovastatin cytotoxicity induced alone or in combination with gefitinib is mediated by apoptosis induction. *(A)* Western blot analysis was used to determine the onset of apoptosis to 10 µM lovastatin, 10 µM gefitinib and their combination through visualization of cleaved Poly ADP-ribose Polymerase (c-PARP), a hallmark of cellular apoptosis. c-PARP was induced with both agents, however, their combination displayed a more rapid and robust response (readily detected in 12 hrs). *(B)* LC3 is a major constituent of the autophagosome, and during autophagy, the cytoplasmic form (LC3-I) is processed and recruited to the autophagosomes where LC3-II is generated. In the treatments outlined above in the SCC25 cells, only the 10 µM gefitinib treatment induced significant conversion to the LC3-II isoform that was not enhanced by its combination with 10 µM lovastatin. *(C)* The hallmark of autophagic activation is the formation of cellular autophagosome punctae containing LC3-II. Fluorescent microscopy employing exogenously expressed LC3-GFP chimeric protein showed that fewer than 2% of cells at 48 hr treatment with 10 µM lovastatin displayed LC3-GFP expression in a punctae-staining pattern resembling putative autophagosomes.

### Statistical Analysis and Evaluation of Therapeutic Interactions

All MTT and ROS data are presented as mean±SD of 6 replicates with each experiment performed at least three times with similar results. One-way ANOVA and *t*-test were performed to determine the differences between control and treatment groups. Statistical analysis was carried out using SPSS version 13.0 (SPSS Inc., Chicago, IL). A *P*-value <0.05 was considered significant.

The combination effect of metformin or lovastatin with gefitinib was evaluated by the Chou-Talalay method using CalcuSyn computer software (Biosoft, Cambridge, GBR). The dose-effect curves of each drug alone, and in combination, were produced by MTT assay. This data was entered into the CalcuSyn software and combination index (CI) values were graphed on fraction affected-CI (Fa-CI) plots. A CI<1 is a synergistic interaction, CI = 1 is additive, and CI>1 is antagonistic.

## Results

### Lovastatin Induces LKB1 and AMPK Activation in SCC Cells

In our recent work, we have demonstrated the ability of lovastatin to induce metabolic stress through the activation of the ISR [18] and inhibition of ligand induced EGFR/AKT activation [19]. Another metabolic stress pathway, induced by LKB1/AMPK activation, has common downstream targets to these pathways [11] (Figure 1A) and the goal of this study was to evaluate the potential of lovastatin to activate LKB1/AMPK and determine their role in regulating lovastatin-induced apoptosis of SCC cells. Employing Western blot analyses of lovastatin treated SCC25 cells (24 hr- 0, 1 10 and 25 µM); we confirmed expression changes of key regulators of the ISR and growth factor withdrawal pathways mediated by the EGFR (Figure 1B). Lovastatin treatment inhibited EGF induced activation of AKT coincident with induction of ATF3, a key regulator of the ISR [5]. CyclinD1 expression is required for cell cycle G1/S transition while p21 protein binds to and inhibits the activity of cyclin complexes, and thus inhibits cell cycle progression at G1 [35]. As previously demonstrated, lovastatin treatment down-regulated cyclinD1 expression and induced p21 expression in SCC25 cells [19]. Similarly, lovastatin inhibited the phosphorylation of 4EBP1 that is required for cap dependant protein translation (Figure 1B). Therefore, lovastatin treatment of SCC25 cells triggers cellular stress.

Due to the potential overlap of the three cellular stress pathways outlined in Figure 1A, we determined the effect of lovastatin treatment on the activity of the LKB1/AMPK pathway. Due to the common disruption of this pathway in tumour cells [29], we confirmed the ability of SCC25 cells to activate AMPK employing well-established pharmaceutical agents targeting AMPK activity. Metformin activates LKB1 by increasing AMP concentration through inhibition of adenylate kinase and subsequent activation of AMPK [31]. AICAR is also an AMPK activator that is a cell permeable mimetic to AMP while compound C competes with ATP for its binding site in the kinase domain of AMPK inhibiting its activation [28]. We treated SCC25 cells with metformin (24 hrs- 0, 1, 5, 10 mM) and the phosphorylation status of LKB1 and AMPK was determined by Western blot analysis employing antibodies recognizing the phosphorylated and activated forms of LKB1 (Ser428) and AMPKα (Thr172) and compared to total protein levels. We found that in response to metformin treatment, SCC25 cells induced LKB1 and AMPKα phosphorylation in a dose-dependent manner (Figure 2A). AICAR treatment (24 hrs- 0, 0.25, 0.5, 1 nM) also induced the phosphorylation of AMPKα in SCC25 cells (Figure 2B). In contrast, treatment of SCC25 cells with compound C (24 hrs- 0, 1, 2, 5 µM) inhibited AMPK activation (Figure 2B). Similar effects on LKB1 and AMPK activation from these three compounds were also demonstrated in SCC9 cells (data not shown).

Taken together, this data demonstrated the ability of both SCC9 and SCC25 cells to activate the LKB1/AMPK pathway. We then tested whether LKB1 and AMPK can be activated by lovastatin in these cell lines. We treated SCC9 and SCC25 cells with 0, 1, 10 and 25 µM lovastatin for 24 hrs. As demonstrated by Western blot analysis, lovastatin treatment of SCC25 (Figure 2C) and SCC9 (Figure 2D) cells induced phosphorylation of both LKB1 and AMPK.

### Lovastatin Affects ATP Levels in SCC Cells

For efficient activation of AMPK both LKB1 activation and AMP binding to AMPK are required [28]. The majority of cellular ATP is generated in mitochondria and deregulated mitochondrial function can affect AMP/ATP ratios [36], [37]. To assess mitochondrial function, we evaluated mitochondrial morphology as well as reactive oxygen species (ROS) generation as early markers of mitochondria dysfunction [36], [37]. SCC25 cells were treated with 10 µM lovastatin for 24 hrs; a condition that we have previously demonstrated was prior to induction of an apoptotic response in these cells. In control cells, cytochrome c was expressed in tube-like structures typical of mitochondrial distribution pattern. In lovastatin treated SCC25 cells, cytochrome c staining was evident in a more punctate staining pattern (Figure 3A). This pattern is indicative of mitochondrial fragmentation, a common feature under conditions of mitochondrial dysfunction and mitochondrial mediated apoptosis [36], [37].

We next evaluated cellular ROS production by using an oxidation-based fluorescence assay and found that ROS production was increased in SCC25 cells following 24 hr 10 µM lovastatin treatment (p<0.05) as above (Figure 3B). A decrease in ATP levels is often associated with an increase in ROS. We then determined cellular ATP levels coincident with 0, 1, 10 and 25 µM lovastatin and as a comparator with 0, 1, 5, 10 mM metformin in SCC25 cells. After 24 hr treatments, the ADP:ATP ratio was determined for each condition tested. As shown in Figure 3C, an increased ADP:ATP ratio, reflective of declining ATP levels and increased AMP levels was observed in a dose dependant manner for both lovastatin and metformin. Thus, decreased ATP production induced by lovastatin is a likely mechanism regulating LKB1 and AMPK activation in SCC cells.

### Deletion of LKB1 in MEFs Inhibits Lovastatin-Induced Cytotoxicity

To determine the potential role of the activation of the LKB1/AMPK pathway as a regulator of lovastatin-induced cytotoxicity, we evaluated this cytotoxic response in wild-type LKB1+/+ murine embryonic fibroblasts (MEFs) compared to LKB1−/− MEFs. As expected, in the LKB1+/+ MEFs both LKB1 and AMPK are expressed while LKB1 expression is absent from LKB1−/− MEFs as visualized by Western blot analysis (Figure 4A and B). Lovastatin treatment (24 hr- 0, 1 10 and 25 µM) induced phosphorylation of both LKB1 and AMPK only in the LKB1+/+ MEFs and were undetected in the LKB1−/− MEFs (due to the lack of expression of LKB1, actin was used as a loading control in the LKB1−/− MEFs). We next determined whether the presence of LKB1 played a role in regulating lovastatin-induced cytotoxicity. We similarly treated LKB1+/+ and LKB1−/− MEFs with a range of lovastatin concentrations of up to 25 µM for 48 and 72 hrs and changes in cell viability were assessed by the MTT assay. As shown in the left panel of Figure 4C, the LKB1+/+ were significantly more sensitive to 48 hr lovastatin treatments than the LKB1−/−MEFs, which showed minimal effect on cell viability with lovastatin treatment at this time-point. However, at the 72 hr time point, the difference in cytotoxicity observed within these two sets of MEFs had dissipated with similar responses observed between the LKB+/+ and LKB1−/− MEFs (Figure 4C).

The nuclear to cytoplasmic shuttling of LKB1 is important for its activation of AMPK [30]. Important to this study, LKB1 is post-translationally modified by the addition of a farnesyl isoprenoid lipid moiety that is a mevalonate pathway metabolite whose depletion by lovastatin treatment may affect its cellular localization [38]. To address this issue, we expressed a green fluorescent protein (GFP)/LKB1 chimeric protein in the LKB1−/− MEFs and observed its localization in control untreated cells and 10 µM lovastatin treated cells for 24 hrs. In the control cells, LKB1 was exclusively localized in the nucleus of expressing LKB1−/− cells, while in the lovastatin treated cells greater than 80% of expressing cells LKB1 was localized in the cytoplasm (Figure 4D) indicating that the activation of LKB1 in these MEFs was associated with its cytoplasmic shuttling. We subsequently confirmed the inability of lovastatin and metformin to activate AMPK in the human SCC cell line HeLa that is deficient in LKB1 expression. Under similar experimental conditions where we demonstrated the ability of these agents to induce AMPK activation in the LKB1 expressing SCC25 and SCC9 cells, lovastatin (24 hr- 0, 1, 10 and 25 µM) and metformin (24 hrs- 0, 1, 5, 10 mM) failed to induce the phosphorylation of AMPK (due to the lack of expression of LKB1, actin was used as a loading control) (Figure 4E).

### Lovastatin but Not Metformin can Enhance the Cytotoxic Effects of Gefitinib in LKB1 Deficient Tumour Cells

An important clinical implication of our previous work was the demonstration that combining statins with EGFR kinase inhibitors like gefitinib (Iressa) and tarceva (Erlotinib) induced synergistic cytotoxicity in SCC and NSCLC. This synergy may be mediated by the ability of statins to trigger these multiple cellular metabolic stress pathways [18], [19], [25]. In this study, we compared the ability of metformin or lovastatin to enhance the cytotoxicity of gefitinib in various LKB1 deficient cell lines including A549 (NSCLC) and HeLa and LKB1 expressing SCC25 and SCC9 cell lines. The goal of this study was to determine if the ability of statins to target multiple stress pathways might overcome the inactivation of LKB1 and still enhance gefitinib cytotoxicity. The four cell lines described above were evaluated by the MTT assay using varying concentrations of metformin (0–20 mM) or lovastatin (0–100 µM) in combination with gefitinib (1–25 µM) as depicted in Figures 5A and 5B. In all cases the metformin and lovastatin treatments were for 72 hrs (24 hr pretreatment) while the addition of gefitinib was for 48 hrs, the sequence of which was determined to be optimal in our previous studies for the combination activity of lovastatin and gefitinib [19], [25]. The combination of metformin and gefitinib induced co-operative cytotoxicity that was limited to the LKB1 expressing cell lines SCC9 and SCC25 with minimal or no co-operativity observed in A549 and HeLa cells that lack LKB1 (Figure 5A). In contrast, as previously published all four of the cell lines tested demonstrated significant co-operative cytotoxicity with combined lovastatin and gefitinib treatments (Figure 5B, data not shown) [19], [25].

To understand the nature of the combination effect, the Chou-Talalay method was used to distinguish between antagonistic, additive and synergistic interactions. The cell lines evaluated were A549 and SCC9. The Combination Index (CI) values of 1 are additive, less than 1 are synergistic and more than 1 are antagonistic and determined by CalcuSyn 2.0 software analysis. In the LKB1 deficient A549 cell line, metformin in combination with 10 µM gefitinib displayed antagonism in combination, while lovastatin in combination with 10 µM gefitinib consistently displayed synergism (Figure 5C). In the SCC9 cell lines similar combinations of metformin or lovastatin with 10 µM gefitinib both displayed synergy in combination (Figure 5D).

Autophagy is a degradative process that results in the breakdown of intracellular material within lysosomes that can be induced by cellular stress pathways such as the ISR, activation of LKB1/AMPK and growth factor withdrawal evaluated in this study [39]. In tumor cells, autophagy is often up regulated to facilitate survival under stress conditions [40]. A number of anticancer therapies including gefitinib can induce autophagy in NSCLC enhancing resistance to this agent [41]. Excessive autophagy, however, can lead to type II programmed cell death, which is morphologically distinct from apoptosis and generally caspase independent [39]. In this study, we evaluated the potential of autophagy to play a role in regulating the cytotoxic synergy of lovastatin in combination with gefitinib in SCC25 cells. To assess the timing of the onset of apoptosis to 10 µM lovastatin, 10 µM gefitinib and their combination, the level of cleaved Poly ADP-ribose Polymerase (c-PARP), a target of caspase activity, was correlated with the potential induction of an apoptotic response (Figure 6A). As single agents, cleaved PARP was detected following 24 hrs of treatment while in their combination; it was readily detected within 12 hrs of treatment with significantly higher levels observed (Figure 6A).

The microtubule-associated protein light chain 3 (LC3) is a major constituent of the autophagosome, and during autophagy, the cytoplasmic form (LC3-I) is processed and recruited to the autophagosomes, where LC3-II is generated by site-specific proteolysis and lipidation near to the C-terminus [39]. The hallmark of autophagic activation is the formation of cellular autophagosome punctae containing LC3-II [39]. In the treatments outlined above in the SCC25 cells, only the 10 µM gefitinib treatment induced significant conversion to the LC3-II isoform that was not enhanced by its combination with 10 µM lovastatin (Figure 6B). Furthermore, exogenous expression of an LC3-GFP chimeric protein showed that fewer than 2% of cells at 48 hr treatment with 10 µM lovastatin displayed LC3-GFP expression in a punctae-staining pattern resembling putative autophagosomes (Figure 6C). Although lovastatin treatment can induce multiple cellular stress pathways, its cytotoxicity induced alone or in combination with gefitinib is mediated by apoptosis induction.

## Discussion

This study is the first demonstration that lovastatin treatment of SCC cells and MEFs induces the LKB1/AMPK metabolic stress pathway. Furthermore, we show that this pathway mediates in part the cytotoxic and apoptotic effects of lovastatin in these cells. Lovastatin induced stress displays the molecular characteristics associated with this response. Lovastatin induced the phosphorylation of LKB1 and AMPK that was associated with changes in mitochondrial function and lower cellular ATP concentrations. Cellular redistribution of LKB1 plays a significant role in the activation of AMPK during energy stress that is mediated by co-factors STRADα and MO25 where STRADα induces its re-localization from the nucleus to the cytoplasm and MO25 stabilizes this interaction stimulating LKB1 activation of AMPK [30]. Lovastatin treatment resulted in this re-localization as demonstrated by the nuclear to cytoplasmic shuttling of the LKB1-GFP chimeric protein expressed in LKB1−/− MEFs. LKB1 knock out MEFs and LKB1 deficient HeLa cells also failed to activate AMPK in response to lovastatin treatment suggesting that its activation is dependant on LKB1 activity. Furthermore, these LKB1−/− MEFs demonstrate an attenuated and delayed cytotoxic response to lovastatin suggesting a role for LKB1/AMPK in regulating this response but that other pathways are also involved.

Of clinical significance, both lovastatin and metformin induce synergistic cytotoxicity in combination with the EGFR inhibitor gefitinib in LKB1 expressing tumour cells, however likely due to its ability to activate multiple metabolic stress pathways; lovastatin also induced synergy with gefitinib in LKB1 deficient tumours. This suggests that while the LKB1/AMPK pathway plays a role in regulating lovastatin-induced cytotoxicity, the ability of lovastatin to affect a number of cellular stress pathways can overcome the lack of LKB1. This property highlights the potential of statins through their pleiotropic effects on a number of cell signaling pathways to have broad therapeutic applications [13], [18], [19].

HMG-CoA reductase and the LKB1/AMPK pathway may be linked due to the potential of mevalonate metabolites to affect cellular and mitochondrial homeostasis. The rate-limiting step of the mevalonate pathway is the conversion of HMG-CoA to mevalonate, which is catalyzed by HMG-CoA reductase [15]. The mevalonate pathway produces various end products that are critical for many different cellular functions including cholesterol, dolichol, ubiquinone, isopentenyladenine, geranylgeranyl pyrophosphate and farnesyl pyrophosphate [15]. Cholesterol is essential in maintaining cellular membrane structure and integrity. Dolichol works as a carrier molecule of oligosaccharides in N-linked protein glycosylation for the production of glycoproteins. Ubiquinone is involved in mitochondrial respiration while isopentenyladenine is an essential substrate for the modification of certain tRNAs [15]. Geranylgeranyl pyrophosphate and farnesyl are common post-translational additions to a wide variety of cellular proteins [42], [43]. These include Ras, nuclear lamins, and many small GTP-binding proteins such as members of the Rab, Rac, and Rho families. These proteins regulate cell proliferation, intracellular trafficking and cell motility and this post-translational modification functions as a membrane anchor critical for their activity [42], [43]. Blockade of the rate-limiting step of the mevalonate pathway by HMG-CoA reductase inhibitors results in decreased levels of mevalonate and its downstream products and, thus, may have significant influences on many critical cellular functions.

Malignant cells appear highly dependent on the sustained availability of the end products of the mevalonate pathway. Deregulated or elevated activity of HMG-CoA reductase has been shown in a range of different tumours [12], [44]. The statin family of drugs is potent inhibitors of HMG-CoA reductase that are widely used as hypercholesterolemia treatments. They bind to the active site of HMG-CoA reductase with up to a 1000x higher affinity than its natural substrate [14]. Recent analyses have demonstrated that statin treatment can directly block tumour cell growth, invasion and metastatic potential both *in vitro* and *in vivo* [13], [45], [46]. Identifying the cellular pathways that regulate lovastatin-induced apoptosis in SCC and other malignant cells may have profound clinical significance.

As cancer therapeutic agents, statins even at high doses (5–10x the hypercholesterolemia dose) did not demonstrate clinical activity in mainly breast, prostate and central nervous system cancer patients in a Phase I clinical trial [47]. Renewed interest in statins has revolved around our work that demonstrated that similar high dose statin treatments can induce tumour specific apoptosis in vitro, especially in SCC [16], [48]. This work led to our initial Phase I study with 25% of the SCC patients treated showing stable disease (3–6 months) utilizing lovastatin as a single agent [17]. The clinical utility of statins will likely require their use with other active agents to enhance their efficacy. The elucidation of the mechanism(s) by which statins induce tumour cell apoptosis will uncover such novel combination-based therapeutic approaches. Thus, we have now identified three previously unrecognized metabolic stress pathways that are modulated by statin treatment; the ISR pathway that is activated by a wide variety of insults [18]; growth factor withdrawal manifested through the inhibition of the EGFR [19], [25]; and the LKB/AMPK pathway that senses ATP depletion outlined in this study. The initial cascades triggered by these pathways function to alleviate the stress; if the stress cannot be overcome apoptosis is induced in these damaged cells [27], [49], [50]. We have also demonstrated that targeted disruption of each of these pathways can attenuate lovastatin-induced apoptosis implicating their roles in regulating this response. Therefore, the ability of statins to modulate the activity of multiple metabolic stress pathways is a novel finding with the potential to regulate statin-induced tumour cell apoptosis.

Due to the importance of the EGFR in the pathogenesis of a variety of epithelial cancers including SCC and NSCLC, clinically relevant inhibitors of this receptor have been developed but have shown only limited activity as single agents in NSCLC patients [10], [51]. Based on our work demonstrating the ability of statin treatments to inhibit EGFR activity [19], [25], we evaluated the potential of statins to augment the cytotoxicity of the clinically relevant EGFR tyrosine kinase inhibitors, gefitinib and tarceva. Lovastatin in combination with gefitinib or tarceva induced robust synergistic cytotoxicity with co-operative inhibition of the EGFR in a number of SCC and NSCLC cell lines [19], [25]. We believe that the robust synergy observed in combinations of statins and these EGFR tyrosine kinase inhibitors is due to statins’ ability to modulate the activity of multiple metabolic stress pathways including AMPK activation. As a result of these studies, we initiated a Phase I study evaluating high dose rosuvastatin and the EGFR inhibitor tarceva (erlotinib) in SCC and NSCLC patients at our institute based on their established superior clinical performance (ClinicalTrials.gov Identifier: NCT00966472) [52]. Therefore by identifying key regulators of statin-induced cytotoxicity, a novel combination therapeutic approach was uncovered and importantly translated into a clinical trial.
